# Serum levels of insulin-like growth factor-I, IGF-binding protein 1 and 3, and insulin and endometrial cancer risk

**DOI:** 10.1038/sj.bjc.6601312

**Published:** 2003-10-28

**Authors:** E Weiderpass, K Brismar, R Bellocco, H Vainio, R Kaaks

**Affiliations:** 1 International Agency for Research on Cancer, 150 Cours Albert Thomas, F-69372 Lyon Cédex 08, France; 2 Department of Medical Epidemiology, Karolinska Institutet, Nobelväg 12A, Box 281, Stockholm S-171 77, Sweden; 3 Department of Molecular Medicine, Endocrine and Diabetes Unit, Karolinska Hospital, Stockholm S-17176, Sweden

**Keywords:** endometrial neoplasms, insulin-like growth factor-I (IGF-I), insulin-like growth factor binding protein-1 (IGFBP-1), insulin-like growth factor binding protein-3 (IGFBP-3), insulin, hormone replacement therapy (HRT)

## Abstract

Insulin-like growth factor-I (IGF-I) and IGF-binding protein-1 and 3 (IGFPB-1, IGFPB-3) are expressed in normal and neoplastic endometrium. Their role and the role of insulin in the aetiology of endometrial cancer, is unclear. We performed a population-based case-control study in Sweden, including 288 endometrial cancer patients and 392 control women and analysed total serum IGF-I, IGFBP-1, IGFBP-3, insulin and BMI levels stratified by disease and hormone replacement therapy status (HRT). Non-parametric statistical tests and logistic regression analyses were performed to assess associations with endometrial cancer. There were no substantial differences between the mean serum levels of IGF-I between cases (115.5, s.d. 61.3) and controls (110.6; s.d. 50.4; Wilcoxon *P*=0.84), or between subgroups of women classified according to other risk factors for endometrial cancer. There were no trends of increasing risk according to quartiles of IGF-I, IGFBP-1, IGFBP-3 and insulin serum levels. There was an increasing risk of endometrial cancer according to the serum levels of IGFBP-1, which was observed only among women who had ever used HRT. Serum IGF-I, IGFBP-1, IGFBP-3 and insulin levels seem unrelated to endometrial cancer risk. Among users of HRT, increasing IGFBP-1 levels seem to increase endometrial cancer risk.

Endometrial cancer has high incidence rates in Western, industrially developed societies. In these countries, obesity has been associated with a 2-to-5-fold increase in endometrial cancer risk in both pre- and postmenopausal women (IARC, 2002) and has been estimated to account for about 40% of endometrial cancer incidence (Bergstorm *et al*, 2001). Apart from excess weight, epidemiological evidence suggests that lack of regular physical activity may also be a risk factor (IARC, 2002).

A major metabolic link between obesity, lack of physical activity, and development of ovarian androgen excess is chronic hyperinsulinaemia. Obesity and physical inactivity lead to insulin resistance, and increased fasting and nonfasting insulin levels. Furthermore, type II diabetes mellitus (Type II DM) – a condition associated with chronic endogenous insulin excess for many years both before and after diagnosis – is a well-established risk factor for endometrial cancer (Persson and Adami, 2002). One previous case–control study showed that risk of endometrial cancer was increased among women (also nondiabetic) who had elevated fasting serum levels of C-peptide, a marker of pancreatic (pro)insulin secretion (Troisi *et al*, 1997).

In addition to insulin, there is evidence that endometrial cancer development is related to alterations in insulin-like growth factor-I (IGF)-I metabolism. Oestrogens increase endometrial cell proliferation by inducing the production of IGF-I in stromal tissue, and it is IGF-I that, in turn, provides the major mitogenic stimulus. Progesterone opposes these effects by inducing the local synthesis of IGFBP-1 (Giudice, 1994; Lee *et al*, 1997) – the most abundant IGF-binding protein in endometrial tissue. Insulin inhibits IGFBP-1 synthesis in liver and other tissue, and this may be one key mechanism through which insulin increases endometrial cancer risk (Brismar *et al*, 1994; Lee *et al*, 1997).

We present here results of a population-based case–control study in Sweden, in which we assessed the relationships of endometrial cancer risk with serum levels of fasting insulin, IGF-I, and IGFBP-1, as well as IGFBP-3, IGF's major binding protein in the circulation. We also explored whether these associations differ in subgroups of women who used exogenous hormones (oral contraceptives (OCs) and hormone replacement therapy (HRT)).

## SUBJECTS AND METHODS

### Study population

Our study included women aged 50–74 years, resident between February 1996 and November 1997 in 12 Swedish counties on the coasts of the Gulf of Bothnia, the Baltic Sea, and the largest Swedish lakes. Women were eligible if they were born in Sweden, had no prior hysterectomy, and had no previous history of cancer. In a component of this study, we have previously analysed serum organochlorine levels and endometrial cancer risk (Weiderpass *et al*, 2000). We assumed that the intake of organochlorine compounds through ingestion of possibly contaminated fish would be higher in these fish-producing counties than in other parts of Sweden.

Women with incident histopathologically confirmed endometrial cancer diagnosed between February 1996 and November 1997 were identified through a network of personnel at the 26 departments of gynaecology/gynaecology–oncology in the study area (one of the departments did not collaborate). The health-care system in Sweden is organised in such a way that people must seek services in the health-care unit/hospital closest to their home. Therefore, it is highly unlikely that cancer cases residing in the study area would be operated outside the study area. A total of 396 cases were reported, approximately 95% of the number expected on the basis of national incidence rates (National Board of Health and Welfare, 1998). Of these, 288 (73%) were contacted before surgery, volunteered to donate blood samples, and subsequently completed the study questionnaire a few months after surgery; 41 patients refused to participate and 67 cases were not approached (due to failure of the medical staff to collect a blood sample before surgery).

Population controls, who were resident in the study area, were randomly selected from a continuously updated population register and frequency matched to cases by 5-year age groups. Controls were not matched to cases by geographic area of residence (county) or any other characteristic. The period of control recruitment coincided with that of the cases, since we sampled and enrolled controls in four phases: the spring of 1996, the fall of 1996, the spring 1997, and the fall 1997. In contrast to the cases, the controls were approached first for questionnaire information, and subsequently asked to donate a fasting blood sample. Of 688 control women selected, 505 (73.4%) responded to the questionnaire, and 438 (63.7%) also agreed to donate blood samples. After exclusion of 46 women because of prior hysterectomy, 392 control women were included in the study.

The self-administered study questionnaire requested information on weight 1 year preceding the interview, height, reproductive history, smoking, physical activity 1 year preceding the interview, medical history (as having had a diagnosis of diabetes mellitus), and use of exogenous hormone (of HRT and OCs), among others. Information about use of HRT and OCs included brand, dosage, date of first and last use of each treatment period, and – for HRT – treatment indication. Recall was aided by picture charts of all brands of HRT and OCs commercially available in Sweden during the years 1960–1995. Use of the same methodology (i.e. questionnaire and picture charts to access history of HRT and OCs use) was tested in previous studies, where it identified differential patterns of endometrial cancer risk and different histopathological features of endometrial tumours according to the type of hormones used and the route of administration (Weiderpass *et al*, 1999a, 1999b and 1999c).

Missing information was supplemented by a telephone interview in approximately 50% of cases and controls.

The Ethical Committee, Uppsala University and the Ethical Committee, Karolinska Institutet, Stockholm, Sweden approved the study design. Only patients who gave informed consent were included in the study.

### Blood sampling

Blood samples from fasting case women were drawn at the hospital departments before surgery or any cancer treatment and from controls at a primary health-care unit or at home. Case patients and control women were requested to fast overnight, for at least 8 h.

### Laboratory analysis

Laboratory analysts in charge of measuring IGF-I and IGFBP-1, IGFBP-3, and insulin were blinded to the case–control status of the samples, as well as other subject characteristics.

#### Total IGF-I

Serum concentrations of IGF-I were determined by RIA after separation of IGFs from IGFBPs by acid ethanol extraction and cryoprecipitation (Bang *et al*, 1991). To minimise the interference of remaining IGFBPs in the acid ethanol extracts, Des(1–3)IGF-I was used as a radioligand. The recovery of unlabelled IGF-I was 95% and the intra- and interassay coefficients of variation were 5 and 11%, respectively. The lowest detectable quantity of IGF-I was 0.01 ng tube^−1^. Crossreactivity with insulin was less than 0.1% and with IGF-II less than 2%.

Serum levels of IGF-I are age dependent, decreasing with age. Thus, IGF-I values were also expressed as standard deviation (s.d.) scores, calculated from the regression of the values of 247 healthy adult subjects (Hilding *et al*, 1995).

#### IGFBP-1

Serum IGFBP-1 concentrations were determined by RIA as described by Povoa *et al* (1986). The intra- and interassay coefficients of variation were 3 and 11%, respectively, and the detection limit was 3.0 *μ*g l^−1^. Crossreactivity with IGFBP-2 and IGFBP-3 was less than 0.5 and 0.05%, respectively. The geometrical mean and range of IGFBP-1 were 34 and 12–91 *μ*g l^−1^ in healthy subjects, aged 20–66 years (Hall *et al*, 1988).

#### IGFBP-3

IGFBP-3 was analysed using a commercial RIA (DSL 6700, Diagnostic System Laboratories, Webster, TX, USA). The mean and normal range was 3.8 and 2.3–5.3 mg l^−1^ in women.

#### Insulin

Serum insulin was measured using an RIA technique (RIA 100, Pharmacia, Uppsala, Sweden). The detection limit was <2 *μ*U ml^−1^. The within-assay CV was 5.8 for a mean value of 11.6 *μ*U ml^−1^ and 5.7 for a mean value of 65.2 *μ*U ml^−1^.

### Statistical analysis

As the distributions of IGF-I, IGFBP-1, IGFBP-3, and insulin measurements were heavily skewed, we used the nonparametric two-sample Wilcoxon's test for unpaired data to conduct unadjusted comparisons of IGF-I, IGFBP-1, IGFBP-3, insulin levels in case and control women and in ever, never, and former users of HRT. Quartiles of the distribution of the IGF-I, IGFBP-1, IGFBP-3, and insulin levels were calculated based on the values assumed from the controls (reference group), and included in the logistic regression analysis as dummy variables.

We first estimated age-adjusted odds ratios (ORs) of IGF-I, IGFBP-1, IGFBP-3, and insulin serum levels, and subsequently included in the logistic regression models variables known or hypothetically associated with endometrial cancer risk, and that may be in the ‘IGF-I, IGFBP-1, IGFBP-3, insulin, and endometrial cancer pathway. These variables were age (as a continuous variable), age at menarche (as a continuous variable), menopausal status (pre- or postmenopausal), body mass index (BMI, that is, weight in kg height^−1^ in m^2^, as a continuous variable), physical activity levels, use of oral contraceptives (ever or never), clinical history of diabetes mellitus and hypertension (self-reported), and use of different HRT (classified according to ever or never exposure to the following compounds: oestrogens without progestins, oestrogens with cyclic addition of progestins, oestrogens with continuous addition of progestins, progestins without oestrogens, oral oestriol, and vaginal use of oestriol, dienoestrol, or oestradiol. Ever users were further classified as ‘current users’, meaning control women who were using HRT at the time of blood sample collection and case women who were using HRT at the time of blood sample collection during the process of endometrial cancer diagnosis).

We analysed the effect of all the variables mentioned above on IGF-1, IGFBPs, and insulin among control women only, to understand which were strong determinants of these endogenous hormone levels among women without cancer. In these analyses, the use of HRT and OCs was the only women's characteristic strongly influencing all endogenous hormones levels of interest (IGF-1, IGFBPs, and insulin). Therefore, here we will also present stratified analysis according to the use of HRT and OCs.

Maximum-likelihood estimates of ORs and 95% confidence intervals were produced using the unconditional logistic regression model procedure in Stata 7 (Breslow and Day, 1980; Stata, 2000).

## RESULTS

Compared to controls, cases were slightly older, had a higher age at menopause, a lower parity, and a greater BMI. Proportionally, more cases than controls reported being nulliparous, having never smoked or used oral contraceptives, having used HRT, having a history of diabetes mellitus or hypertension, and being extremely sedentary (Table 1

**Table 1:** Selected characteristics of endometrial cancer patients and control women

		**Cases**	**Controls**
	**Cases/controls^a^**	**Mean (s.d.)**	
Age (years)	288/392	64.7 (7.2)	63.0 (7.0)
Age at menopause^b^	258/363	51.2 (3.3)	50.3 (3.7)
Parity (all women)	288/381	1.9 (1.2)	2.2 (1.2)
Age at last birth^c^	288/381	30.0 (5.5)	30.0 (5.5)
Body mass index^d^	288/291	27.6 (5.0)	25.9 (4.4)
			
		**Cases**	**Controls**
		**Proportions**	
Premenopausal	288/392	7.7%	6.4%
Nulliparous	288/381	14.6%	9.4%
Ever smoked	288/392	29.2%	39.5%
Ever used OCs	288/392	71 (24.7%)	154 (39.3%)
Ever used HRT^e^	288/392	150 (52.1%)	166 (42.3%)
Diabetes mellitus^f^	288/392	10.4%	3.1%
Hypertension^f^	281/391	35%	24%
Extreme sedentarism^g^	208/366	20 (9.6%)	4 (1.1%)

a Number of study subjects for whom data are available for each variable.

b Excluding 22 cases and 25 controls who were premenopausal.

c Among parous.

d Weight in kg height^−1^ in m^2^.

e HRT=hormone replacement therapy. Women may have used more than one kind of oestrogen replacement.

f Self-reported.

g At 1 year before study enrolment in a scale of physical activity ranging from 0 to 4.

).

The correlation coefficients between the different endogenous hormones studied did not differ substantially from those calculated separately for cancer patients and controls (Table 2

**Table 2:** Pearson's correlation coefficients between the various hormone levels in the entire study population (endometrial cancer cases and control women)

	**INSULIN (ng ml^−1^)**	**IGFBP-1 (ng ml^−1^)**	**IGF-I (ng ml^−1^)**
IGFBP-1 (ng ml^−1^)	−0.22	1.0	
IGF-I (ng ml^−1^)	0.04	−0.09	1.0
IGFBP-3 (ng ml^−1^)	0.09	−0.18	0.35

a The correlation coefficients calculated separately for endometrial cancer cases and control women were not substantially different from the coefficients presented above (cases and controls together).

).

Table 3

**Table 3:** Distribution of IGF-I, IGFBP-1, IGFBP-3, and insulin among endometrial cancer cases and control women

	**Cases (*n*=288)**				**Controls (*n*=392)**				
	***n***	**Mean (s.d.)**	**Median**	**Range**	***n***	**Mean (s.d.)**	**Median**	**Range**	*P*-value*
IGF-I (ng ml^−1^)	274	115.5 (61.3)	103.0	22–403	313	110.6 (50.4)	101.0	18–373	0.8422
IGFBP-1 (ng ml^−1^)	274	32.2 (20.4)	28.2	1.9–101	313	28.9 (19.1)	24.6	2.7–112	0.0430
IGFBP-3 (ng ml^−1^)	257	6.1 (2.3)	5.7	0.1–12.4	286	6.1 (2.3)	6.0	0.9–12.4	0.6192
Insulin (ng ml^−1^)	260	14.0 (13.9)	10.0	2.0–119.0	296	15.3 (16.7)	10.0	2.0–149.0	0.6351

* *P*-value for the difference between cases and controls (within lines).

presents the number of patients having samples analysed for IGF-I, IGFBP-1, IGFBP-3, and insulin, showing means (and s.d.), medians, and range of results among endometrial cancer cases and control women. Overall, IGFBP-1 levels were higher among the cancer cases than among control women. For the other endogenous hormone levels studied, the differences between cases and controls were not statistically significant.

We further analysed separately the levels of endogenous hormones according to use of HRT (ever, never, and former use) and oral contraceptives (ever and never) (Table 4

**Table 4:** Distribution of IGF-I, IGFBP-1, IGFBP-3, in subgroups of endometrial cancer cases and control women, classified according to use of HRT and OCs

	**Cases (*n*=288)**					**Controls (*n*=392)**					
	***n***	**Mean (s.d.)**	**Median**	*P*-value*	**Range**	***n***	**Mean (s.d.)**	**Median**	*P*-value*	**Range**	*P*-value**
*IGF-I (ng ml*^−*1*^)											
HRT never used	129	107.9 (55.7)	98.0	0.0584	27–333	203	100.8 (45.5)	94.0	0.000	18–290	0.5052
HRT ever used	145	122.2 (65.3)	108.0		22–403	110	128.8 (54.2)	118.5		57–373	0.1212
HRT former user	116	122.8 (62.5)	111.0	0.0382	22–381	25	129.2 (56.2)	109.0	0.0168	57–278	0.5708
OC never used	204	112.8 (61.3)	99.0	0.1150	27–403	203	105.8 (53.6)	94.0	0.0007	18–373	0.3703
OC ever used	70	123.4 (61.0)	110.0		22–333	110	119.4 (42.7)	113.5		41–290	0.8973

*IGFBP-1 (ng ml*^−*1*^)											
HRT never used	129	33.1 (21.6)	26.9	0.6611	1.9–98.6	203	32.3 (19.4)	30.9	0.000	4.0–112.0	0.9425
HRT ever used	145	31.3 (19.4)	27.2		3.2–101.0	110	22.4 (16.6)	17.1		2.7–82.9	0.0000
HRT former user	116	31.4 (20.2)	27.5	0.6062	3.9–101	25	22.7 (18.0)	13.6	0.0078	2.7–62.2	0.0312
OC never used	204	32.2 (20.2)	28.2	0.9484	3.9–101	203	32.4 (20.1)	30.7	0.0000	2.7–112.0	0.8821
OC ever used	70	32.2 (21.2)	29.0		1.9–95.4	110	22.6 (15.1)	20.6		4.0–79.7	0.0019

*IGFBP-3 (ng ml*^−*1*^)											
HRT never used	120	6.1 (2.3)	5.7	0.8092	0.1–11.9	177	5.8 (2.2)	5.4	0.0003	1.6–11.5	0.3469
HRT ever used	137	6.1 (2.3)	5.7		2.2–12.4	109	6.8 (2.4)	6.4		0.9–12.4	0.0179
HRT former user	112	6.1 (2.3)	5.7	0.9415	2.2–12.4	25	6.8 (2.4)	7.3	0.0154	0.9–11.2	0.0950
OC never used	192	6.1 (2.2)	5.7	0.6191	1.8–12.4	187	6.0 (2.4)	5.6	0.0500	0.9–12.4	0.6036
OC ever used	65	6.0 (2.4)	5.6		0.1–11.4	99	6.4 (2.2)	6.4		1.6–12.0	0.1399

*Insulin (ng ml*^−*1*^)											
HRT never used	120	14.8 (13.4)	10.0	0.0727	7.0–81.0	187	11.0 (9.2)	9.0	0.0000	2.0–64.0	0.0024
HRT ever used	140	13.2 (14.2)	9.0		2.0–119.0	109	22.6 (22.9)	16.0		3.0–149.0	0.0000
HRT former user	115	12.3 (11.7)	9.9	0.0773	2.0–71.0	25	22.3 (22.6)	16.0	0.0007	5.0–90.0	0.0081
OC never used	194	14.2 (14.1)	10.0	0.5930	2.0–119.0	194	15.6 (18.7)	10.0	0.5392	2.0–149.0	0.9573
OC ever used	66	13.3 (12.9)	10.0		2.0–81.0	102	14.6 (11.9)	10.0		3.0–71.0	0.3581

HRT=hormone replacement therapy; OC-oral contraceptives.

* *P*-value for the difference within categories in each column (i.e. comparing cases who used HRT with cases who never used HRT or who were former users of HRT, or ever *vs* never users of OCs within cases and within controls).

** *P*-value for the difference between cases and controls (within lines).

). Regardless of control status, women who used HRT either at the time of the blood donation or previously presented significantly higher IGF-I levels than women who never used HRT, and a similar difference was observed between women who did or who did not use OCs. The exclusion of women who were using HRT at the time of blood donation from the analysis did not alter these differences substantially. Likewise, but among the control women only, those never users of HRT or OCs had lower levels of IGFBP-3 and higher levels of IGFBP-1 than HRT users. Moreover, only the HRT users had lower levels of insulin.

The higher levels of IGFBP-1 among endometrial cancer cases were restricted to those women who had used HRT and/or OC. Among HRT or OC users, cases also had lower IGFBP-3 levels than controls, although the difference did not reach statistical significance. Among never users of HRT, insulin levels were higher among cases compared to controls, whereas among HRT users the opposite was observed: cases had lower insulin levels than controls. In all subgroups of HRT and/or OC use, cases had higher BMI than controls, although cases who had used HRT had slightly lower BMI than cases who never used HRT (Table 4).

Overall, all subgroups of HRT and OC users combined, there was no significant trend of increasing or decreasing the risk of endometrial cancer over quartiles of the distribution of IGF-I, IGFBP-1, IGFBP-3, and insulin serum (Table 5

**Table 5:** Serum levels of IGF-I, IGFBP-1, IGFBP-3, insulin, and risk of endometrial cancer

		Quartiles of the distribution among control women*			
		**2**	**3**	**4**	
Age-adjusted and multivariate models**	**1 (reference)**	OR (95 CI%)*	OR (95 CI%)*	OR (95 CI%)*	***P* trend**
*IGF-I*					
Model A	1.0	0.63 (0.39–1.01)	0.84 (0.53–1.33)	0.98 (0.62–1.53)	0.85
Model B	1.0	0.51 (0.29–0.89)	0.66 (0.38–1.17)	0.89 (0.52–1.53)	0.86
Model C	1.0	0.59 (0.32–1.10)	0.82 (0.44–1.54)	0.86 (0.46–1.58)	0.78

*IGFBP-1*					
Model A	1.0	0.96 (0.59–1.56)	1.29 (0.81–2.07)	1.43 (0.90–2.27)	0.07
Model B	1.0	1.16 (0.64–2.11)	1.50 (0.85–2.63)	1.63 (0.90–2.94)	0.07
Model C	1.0	0.91 (0.45–1.81)	1.21 (0.63–2.35)	1.09 (0.54–2.20)	0.85

*IGFBP-3*					
Model A	1.0	0.93 (0.58–1.50)	0.76 (0.47–1.24)	0.90 (0.55–1.46)	0.51
Model B	1.0	0.99 (0.56–1.74)	0.81 (0.45–1.48)	1.08 (0.60–1.92)	0.95
Model C	1.0	0.77 (0.41–1.46)	0.80 (0.41–1.56)	1.22 (0.63–2.36)	0.51

*Insulin*					
Model A	1.0	1.18 (0.72–1.95)	1.09 (0.65–1.83)	1.04 (0.62–1.75)	0.94
Model B	1.0	1.13 (0.62–2.08)	0.77 (0.40–1.51)	0.64 (0.32–1.24)	0.07
Model C	1.0	1.24 (0.62–2.50)	0.87 (0.40–1.90)	0.72 (0.33–1.58)	0.19

OR=odds ratio.

* The first quartile is always considered as a reference category (1.0). The quartiles are: IGF-I (ng ml^−1^): <77.5, 77.5–100, 101–134, and >134. IGFBP-1 (ng ml^−1^): <14.2, 14.2–24.5, 24.6–38.6, and >38.6. IGFBP-3 (ng ml^−1^): <4.5, 4.5–5.9, 6–7.6, and >7.6. Insulin (mg ml^−1^): <6, 6–9.9, 10–16.9, and >16.9.

** The multivariate models include indicators for: Model (A) age, Model (B) age, BMI, diabetes mellitus, and physical activity, Model (C) as in Model (B), adding menopausal status, different types of hormone replacement therapy and oral contraceptives.

). Furthermore, results from the analysis including mutual adjustment IGFBP-1 and insulin serum levels (i.e. results for IGFBP-1 adjusted for insulin levels and results from insulin levels adjusted for IGFBP-1 levels) did not differ meaningfully from the analysis without such mutual adjustment (data not shown).

Although globally there were no clear associations of endometrial cancer risk with levels of the various peptides, certain differences are shown in Table 6

**Table 6:** Serum levels of IGF-I, IGFBP-1, IGFBP-3, insulin, and risk of endometrial cancer, according to use of HRT (ever or never used during lifetime)

	**Quartiles (among controls)**				
	**1**	**2**	**3**	**4**	
Age-adjusted and multivariate models**	**OR (95 CI%)**	**OR (95 CI%)**	**OR (95 CI%)**	**OR (95 CI%)**	***P* trend**
IGF-I (*P* interaction IGF1/HRT=0.22)					
*HRT ever users*					
Cases/controls	39/13	28/30	29/27	49/40	
Model A	1.0	0.32 (0.014–0.72)	0.39 (0.17–0.89)	0.45 (0.21–0.97)	0.18
Model B	1.0	0.24 (0.09–0.64)	0.36 (0.13–0.96)	0.38 (0.16–0.92)	0.17
Model C	1.0	0.46 (0.14–1.48)	0.62 (0.19–2.03)	0.43 (0.15–1.23)	0.19

*HRT never users*					
Cases/controls	45/65	23/48	34/50	27/40	
Age (years)	1.0	0.73 (0.39–1.36)	1.08 (0.60–1.95)	1.08 (0.57–2.03)	0.65
Age, BMI, DM, PA	1.0	0.54 (0.24–1.18)	0.78 (0.36–1.67)	1.22 (0.55–2.69)	0.66
Age, BMI, DM, PA, MP, OC	1.0	0.59 (0.26–1.32)	0.98 (0.44–2.20)	1.44 (0.62–3.31)	0.38

IGFBP-1 (*P* interaction BP1/HRT=0.02)					
*HRT ever users*					
Cases/controls	31/43	30/31	40/19	44/17	
Model A	1.0	1.30 (0.66–2.58)	2.69 (1.29–5.60)	3.29 (1.56–6.93)	<0.0001
Model B	1.0	1.50 (0.64–3.51)	3.76 (1.52–9.30)	4.12 (1.60–10.61)	0.001
Model C	1.0	1.58 (0.56–4.44)	6.24 (1.80–21.66)	3.84 (1.10–13.42)	0.012

*HRT never users*					
Cases/controls	26/37	23/45	37/59	43/62	
Model A	1.0	0.72 (0.35–1.47)	0.86 (0.44–1.64)	0.94 (0.49–1.78)	0.94
Model B	1.0	0.85 (0.35–2.03)	0.82 (0.37–1.81)	0.94 (0.40–2.19)	0.89
Model D	1.0	0.65 (0.25–1.72)	0.53 (0.22–1.27)	0.54 (0.21–1.38)	0.19

IGFBP-3 (*P* interaction BP3/HRT=0.09)					
*HRT ever users*					
Cases/controls	33/15	41/27	28/29	35/38	
Model A	1.0	0.66 (0.30–1.45)	0.43 (0.19–0.96)	0.43 (0.20–0.92)	0.02
Model B	1.0	0.85 (0.33–2.20)	0.49 (0.18–1.30)	0.55 (0.22–1.38)	0.12
Model C	1.0	0.97 (0.29–3.24)	0.56 (0.17–1.88)	0.91 (0.29–2.94)	0.73

*HRT never users*					
Cases/controls	33/51	31/49	28/45	28/32	
Model A	1.0	0.97 (0.52–1.83)	0.97 (0.51–1.86)	1.33 (0.68–2.61)	0.46
Model B	1.0	0.86 (0.40–1.88)	0.78 (0.34–1.78)	1.54 (0.68–3.52)	0.42
Model D	1.0	0.68 (0.30–1.56)	0.86 (0.36–2.07)	1.53 (0.64–3.64)	0.32

Insulin (*P* interaction ins/HRT=0.000)					
*HRT ever users*					
Cases/controls	29/13	44/25	38/20	29/51	
Model A	1.0	0.75 (0.33–1.71)	0.82 (0.35–1.93)	0.24 (0.11–0.54)	<0.0001
Model B	1.0	0.72 (0.26–1.97)	0.55 (0.18–1.66)	0.13 (0.04–0.40)	<0.0001
Model C	1.0	0.72 (0.18–2.83)	0.44 (0.07–1.98)	0.11 (0.03–0.49)	<0.0001

*HRT never users*					
Cases/controls	15/43	39/62	29/56	37/26	
Model A	1.0	1.79 (0.88–3.65)	1.47 (0.70–3.08)	3.97 (1.82–8.66)	0.002
Model B	1.0	1.52 (0.65–3.56)	0.88 (0.34–2.30)	2.18 (0.81–5.84)	0.29
Model D	1.0	1.60 (0.64–3.98)	1.07 (0.38–3.03)	2.44 (0.85–7.07)	0.18

HRT=hormone replacement therapy; OC=oral contraceptives.

** The multivariate models include indicators for: Model (A) age. Model (B) age, body mass index (BMI), diabetes mellitus, and physical activity. Model (C) same as Model (B) adding menopausal status, types of HRT used (unopposed oestrogens, cyclic combined, continuous combined), and use of OCs. Model (D) same as Model (C) with or without HRT.

by HRT status. Among HRT users, higher IGF-I and IGFBP-3 levels were associated with a (nonsignificant) decreased risk of endometrial cancer, while among never users there was no such association. Furthermore, among HRT users increasing levels of IGFBP-1 and lower levels of insulin were associated with increased endometrial cancer risk, while among nonusers the risk was not associated with IGFBP-1 and directly associated with insulin. The differences between HRT users and nonusers in the relationships of risk with IGFBP-1 and insulin were statistically significant (*P* for interaction=0.02 for IGFBP-1 and *P*<0.000 for insulin).

We found no clear evidence of an interaction between the use of oral contraceptives levels of IGF-I, IGFBPs, and insulin (Table 7

**Table 7:** Serum levels of IGF-I, IGFBP-1, IGFBP-3, insulin, and risk of endometrial cancer, according to use of oral contraceptives (OC, ever ,or never used during lifetime)

	**OR (95 CI%)^a^**				
	**1**	**2**	**3**	**4**	***P* trend**
Quartiles and IGF-I (*P* interaction IGF1/OC=0.17)					
*Ever used OC*					
Cases/controls: 70/110					
Age (years)	1.0	0.30 (0.11–0.81)	0.33 (0.13–0.86)	0.58 (0.23–1.49)	0.58
Age, diabetes mellitus, HRT types, BMI	1.0	0.22 (0.07–0.68)	0.28 (0.10–0.82)	0.40 (0.14–1.18)	0.63

*Never used OC*					
Cases/controls: 204/203					
Age (years)	1.0	0.78 (0.45–1.35)	1.18 (0.68–2.05)	1.07 (0.63–1.80)	0.58
Age, diabetes mellitus, HRT types, BMI	1.0	0.70 (0.39–1.25)	1.09 (0.61–1.94)	0.86 (0.49–1.52)	0.74

IGFBP-1 (*P* interaction BP1/OC=0.08)					
*Ever used OC*					
Cases/controls: 70/110					
Age (years)	1.0	0.98 (0.41–2.39)	1.79 (0.76–4.20)	3.26 (1.39–7.68)	0.004
Age, diabetes mellitus, HRT types, BMI	1.0	0.97 (0.35–2.74)	1.80 (0.67–4.84)	5.24 (1.90–14.48)	0.99

*Never used OC*					
Cases/controls: 204/203					
Age (years)	1.0	0.86 (0.47–1.60)	1.03 (0.58–1.85)	0.98 (0.55–1.73)	0.89
Age, diabetes mellitus, HRT types, BMI	1.0	1.06 (0.55–2.04)	1.38 (0.74–2.56)	1.50 (0.78–2.88)	0.43

IGFBP-3 (*P* interaction BP3/OC=0.19)					
*Ever used OC*					
Cases/controls: 65/99					
Age (years)	1.0	0.54 (0.21–1.39)	0.52 (0.21–1.27)	0.40 (0.15–1.01)	0.06
Age, diabetes mellitus, HRT types, BMI	1.0	0.49 (0.17–1.43)	0.44 (0.16–1.22)	0.20 (0.06–0.62)	0.99

*Never used OC*					
Cases/controls: 192/187					
Age (years)	1.0	1.08 (0.62–1.86)	0.88 (0.49–1.59)	1.24 (0.70–2.22)	0.62
Age, diabetes mellitus, HRT types, BMI	1.0	0.93 (0.52–1.66)	0.79 (0.42–1.46)	1.26 (0.69–2.30)	0.53

Insulin (*P* interaction ins/OC=0.82)					
*Ever used OC*					
Cases/controls: 66/102					
Age (years)	1.0	0.94 (0.37–2.37)	1.02 (0.40–2.62)	0.68 (0.26–1.79)	0.47
Age, diabetes mellitus, HRT-types, BMI	1.0	0.87 (0.31–2.49)	0.97 (0.32–2.91)	0.38 (0.12–1.22)	0.16

*Never used OC*					
Cases/controls: 194/194					
Age (years)	1.0	1.32 (0.72–2.39)	1.12 (0.60–2.08)	1.27 (0.68–2.36)	0.67
Age, diabetes mellitus, HRT-types, BMI	1.0	1.12 (0.58–2.16)	0.76 (0.37–1.56)	0.76 (0.36–1.57)	0.17

HRT=hormone replacement therapy.

a Odds ratios and 95% confidence intervals for models including age only, and age, history of diabetes mellitus, use of different HRT types and body mass index (BMI).

). Women in the two highest quartiles of the IGFBP-1 distribution who used OCs were at increased endometrial cancer risk, while no clear association was observed among those who never used OCs (*P* for interaction=0.08). There was no indication of any differential effect of insulin according to the use of oral contraceptives (*P* for interaction=0.82).

## DISCUSSION

This first large case–control study on endometrial cancer risk in relation to serum levels of IGF-I and IGF-binding proteins-1 and -3, globally showed no association of risk with any of these peptides. Furthermore, risk globally appeared not to be associated with serum insulin levels. Other case–control differences in our study in relation to age, age at menopause, parity, BMI, smoking history use of OCs and HRT, history of diabetes, hypertension, and sedentarism are fully in line with known epidemiological associations (Persson and Adami, 2002).

Considerable evidence suggests that these relationships of risk with lifestyle may be mediated by alterations in the metabolism of endogenous sex steroids. Endometrial cancer risk is increased among both pre- and postmenopausal women who have elevated plasma androstenedione and testosterone, and among postmenopausal women with increased levels of circulating oestrone and estradiol (Persson and Adami, 2002).

Given the increased risk of endometrial cancer among type II diabetics and obese women, and given the results from the previous case–control study by Troisi *et al* (1997), we anticipated that elevated insulin would have been related to higher endometrial cancer risk. We have postulated a number of mechanisms through which this increase might occur (Kaaks *et al*, 2002).

The anticipated direct association of risk with serum insulin levels was clearly present only when we restricted our statistical analyses to women who never used any oestrogen or oestrogen plus progestogen HRT. In this subgroup, we also observed an inverse association with levels of IGFBP-1, but only after multiple statistical adjustments. The findings of this subgroup analysis are fully in line with observations from a pooled study of prospective cohorts in the USA (New York), Northern Sweden (Umeå), and Italy (Milan), where an approximate five-fold increase in risk was observed for women in the highest quintile of C-peptide (Lukanova *et al*, in press). This prospective study was based on women who had not been using HRT during the 6 months (or more) preceding blood donation.

Our stratification of analyses by HRT users and nonusers was motivated by the suspicion that current or recent use of HRT might have altered levels of circulating insulin, IGF-I, or IGFBPs. We observed more elevated levels of IGF-I among HRT users, as well as among users of OCs, in both the case and the control groups. This observation might seem at variance with findings from previous studies, where (current) use of exogenous oestrogens for OCs or HRT was generally associated with a decrease in circulating IGF-I levels. A difference between those studies and ours, however, is that ours included only ex-users of exogenous hormones (though including recent use), whereas the previous studies were generally on women still using OCs or HRT at the time of blood donation. Among controls only, our study showed lower levels of insulin and IGFBP-3 and higher levels of IGFBP-1, among the nonusers of HRT.

Unexpectedly, the risk showed an inverse association with levels of insulin and a strong direct association with levels of IGFBP-1, among women who were or had been regularly using HRT. We have no clear explanation for these inverse findings, compared to our study hypotheses. Our study was carefully designed as a population-based case–control study with detailed questionnaire information on pre-existing illnesses and use of medications, and the contrasting findings could not be explained by confounding by history of diabetes mellitus, physical activity levels, menopausal status, or type of exogenous hormones preparations used.

General concerns in case–control studies on endogenous hormones and cancer risk are possible selection bias, or bias that may be caused by differences between the case and control groups in methods for blood collection, processing, and storage. Selection bias could have occurred if nonparticipation in the study was related differently to insulin, IGF-I, or IGFBP-1 levels among eligible cases and controls. Among cases, the principal reason for nonparticipation was the failure of the hospital staff to collect blood samples, a reflection of the characteristics of the medical personnel and not of the patients.

Another theoretical source of bias would be influences of a tumour on circulating levels of IGF-I, IGFBPs, or insulin. IGF-I and IGFBPs are produced not only in the liver – by far the major source of these peptides in the circulation – but also by most other tissues, including the endometrium. Circulating levels of IGF-I and IGFBP-3 are relatively high, however (about 500–1000 times the concentration of insulin, for example), and it is very unlikely that increased synthesis of IGF-I or IGFBP-3 by tumours would substantially alter circulating levels. Nevertheless, one specific IGF-binding protein known to be produced in excess by some tumour types (e.g. colorectum, prostate) is IGFBP-2, and this may lead to some increase in circulating levels (which are much lower than those of IGF-I and IGFBP-3). However, this binding protein was not measured in the present study.

With respect to the laboratory assays, analysts were blinded to the case or control status of the samples, and hence could not have led to any systematic observation bias. However, one possible source of bias that could explain case–control differences in the levels of insulin as well as IGFBP-1 would be the differences in fasting *vs* nonfasting conditions of cases and controls at the time of blood sampling as (IGFBP-1 levels drop acutely as insulin rises, after food consumption). Another possibility would have been an increase in IGFBP-1 levels due to elevated cortisol, which stimulates hepatic IGFBP-1 synthesis, and which might reflect greater psychological stress among the cancer patients, for example. While such factors might have explained a global case–control difference in the levels of these hormones, it is less evident how they would have led to case–control differences in the relationship of risk with hormone levels within subgroups of HRT users and nonusers separately. However, we cannot exclude the play of chance in our findings, particularly in small subgroups such as HRT users.

Globally, risk was not associated with levels of circulating levels of IGF-I. Over 80% of IGF-I in the circulation originates from the liver. The main physiological stimulus for hepatic IGF-I synthesis is growth hormone. In endometrial tissue, however, oestrogens provide the main stimulus for IGF-I synthesis. Given these differences in physiology, it is quite possible that, contrary to several other forms of cancer (Kaaks and Lukanova, 2001), endometrial cancer risk is relatively independent of circulating IGF-I levels. In some other, small case–control studies, cases were found to have lower levels of IGF-I. Among HRT users, we observed an increased risk of endometrial cancer among women in the lowest quartile level of IGF-I (*P* for trend=0.23), which remained after adjustment for BMI.

In conclusion, our study does not show any evidence of an overall association between endometrial cancer risk and serum levels of IGF-1, IGFBP-1, IGFBP-3, and insulin. Our suggestive finding of an association between IGFBP-1 levels and endometrial cancer risk among women who used HRT needs confirmation by a study with greater statistical power to detect weak associations.
